# AMBIT RESTful web services: an implementation of the OpenTox application programming interface

**DOI:** 10.1186/1758-2946-3-18

**Published:** 2011-05-16

**Authors:** Nina Jeliazkova, Vedrin Jeliazkov

**Affiliations:** 1 Ideaconsult Ltd., Angel Kanchev Str 4, Sofia 1000, Bulgaria

## Abstract

The AMBIT web services package is one of the several existing independent implementations of the OpenTox Application Programming Interface and is built according to the principles of the Representational State Transfer (REST) architecture. The Open Source Predictive Toxicology Framework, developed by the partners in the EC FP7 OpenTox project, aims at providing a unified access to toxicity data and predictive models, as well as validation procedures. This is achieved by i) an information model, based on a common OWL-DL ontology ii) links to related ontologies; iii) data and algorithms, available through a standardized REST web services interface, where every compound, data set or predictive method has a unique web address, used to retrieve its Resource Description Framework (RDF) representation, or initiate the associated calculations.

The AMBIT web services package has been developed as an extension of AMBIT modules, adding the ability to create (Quantitative) Structure-Activity Relationship (QSAR) models and providing an OpenTox API compliant interface. The representation of data and processing resources in W3C Resource Description Framework facilitates integrating the resources as Linked Data. By uploading datasets with chemical structures and arbitrary set of properties, they become automatically available online in several formats. The services provide unified interfaces to several descriptor calculation, machine learning and similarity searching algorithms, as well as to applicability domain and toxicity prediction models. All Toxtree modules for predicting the toxicological hazard of chemical compounds are also integrated within this package. The complexity and diversity of the processing is reduced to the simple paradigm "read data from a web address, perform processing, write to a web address". The online service allows to easily run predictions, without installing any software, as well to share online datasets and models. The downloadable web application allows researchers to setup an arbitrary number of service instances for specific purposes and at suitable locations. These services could be used as a distributed framework for processing of resource-intensive tasks and data sharing or in a fully independent way, according to the specific needs. The advantage of exposing the functionality via the OpenTox API is seamless interoperability, not only within a single web application, but also in a network of distributed services. Last, but not least, the services provide a basis for building web mashups, end user applications with friendly GUIs, as well as embedding the functionalities in existing workflow systems.

## Background

Most of the common tasks in toxicity prediction consist of several typical steps, such as access to datasets, descriptor calculation and validation procedures. Usually, the components that implement these steps are developed from scratch for every new predictive application and this often leads to undesirable duplication of effort and/or lack of interoperability. The availability of a universal set of interoperable components could facilitate the implementation of new specialized applications that combine algorithms in the most appropriate way and allow fast and rigorous model development and testing.

The OpenTox framework [1] is being built as a collaborative effort by the partners in the OpenTox EC FP7 project, and is an attempt to design and implement a framework of web accessible components, solving common tasks in chemical properties prediction. The design objectives were to build a component based system, independent of programming languages and operating systems, where the components could interoperate between themselves and with external software packages, being able to aggregate data from different sources and staying open for future extensions. OpenTox made two major technological choices in order to keep the developments within these constraints: (i) the REpresentational State Transfer (REST) software architecture style allowing platform and programming language independence and facilitating the implementation of new data and processing components; (ii) a formally defined common information model, based on the W3C Resource Description Framework (RDF) [2] and communication through well-defined interfaces ensuring interoperability of the web components.

REST is a software architecture style for network based applications, defined by Roy T. Fielding by analyzing the properties of the World Wide Web and other network architectures, and deriving the architectural constraints that made the WWW successful [3]. There is a plethora of information on RESTful design principles [4], development frameworks and examples. The REST architecture can be briefly summarized as Resource Oriented and the essential architectural constraints are as follows. Every important information entity or collection of entities is considered a resource and is named and addressable (i.e. its content can be retrieved by its address) and supports limited number of operations (e.g. read and write). Any information entity or collection of entities could be considered a resource. A resource may return its content in different formats. The content is regarded as resource "representation". Some operations are safe (have no side effects - e.g. reading a resource) and idempotent (have same effect if executed multiple times), while others are not (e.g. creating new resources). The RESTful API design includes a specification of the allowed representation formats for each resource/operation pair. Another important design constraint is the usage of hyperlinks. It is considered good practice if all resources could be reached via a single or minimum number of entry points. Thus, the representation of a resource should return links to the related resources.

The REST style web services became a popular alternative of SOAP based services and they are considered lighter and easier to use. Contrary to the established WS-* [5] standards, there are currently no standards for RESTful applications, but merely design guides. While the most widely deployed REST applications use the HTTP protocol (and therefore HTTP URIs as identifiers, HTTP methods as operations, and MIME types to specify representation formats), the architecture itself is protocol independent, therefore REST systems that do not use the HTTP protocol could exist, and vice versa. A RESTful application is characterized by complying with the architectural constraints, which are selected to ensure a set of particular properties of a distributed system. It is worthwhile to recall that the REST architecture is envisioned to be a collection of independently deployed and interacting distributed software entities, much like as there are millions of independent web servers, which constitute the WWW. Multiple independent and interacting deployments, is also the intended usage of the OpenTox REST API and AMBIT services as one of its implementations, rather than being a web application made available by a single authority or service provider.

The design of a REST system, based on the HTTP protocol, starts by identifying the domain objects, followed by mapping the objects to resources and defining identifiers (URI patterns) and operations (HTTP verbs) on each resource. In the case of OpenTox, the minimum set of domain objects, identified collaboratively by the partners [1], consists of chemical compounds, properties of chemical compounds, datasets of chemical compounds and their properties (measured or calculated), algorithms (including descriptor calculation, regression, classification, structural alerts, quantum chemistry algorithms, etc.), predictive models (e.g. a model, obtained by applying a machine learning algorithm to a training dataset), validation algorithms, and reports. In addition, tasks are introduced as special resources to allow representation and handling of long running asynchronous jobs. Every resource is identified by a unique web address, following an agreed pattern, specific to the resource type (e.g./algorithm/{id} for algorithms,/compound/{id} for compounds, etc.). The resources can be created (HTTP POST), updated (HTTP PUT) and deleted (HTTP DELETE), or their representations retrieved (HTTP GET). While there are diverging opinions whether POST or PUT should be used for creating resources in a REST application, our view (supported by [3]) is that this issue is irrelevant for the characterisation of a system as RESTful, as the design goals of the REST software architecture style (scalability, statelessness, cacheability, uniformity) are not violated by either choice. The particular choice of using POST for creating subordinate resources is a pragmatic one, as it is supported by popular REST frameworks, the HTTP protocol specification [6], and the Atom Publishing Protocol [7], which is often cited as a reference implementation of a REST system. Two additional features of POST's standard defined behaviour have been also accounted for as desirable properties in our design: (i) non-idempotent, meaning that multiple identical requests would probably result in the creation of separate subordinate resources with identical information [4], and (ii) the URIs of the newly created resources are determined by the server, rather than specified by the client. On the other hand, updates of existing resources (PUT) require the client to specify the resource URI, again in full compliance with the HTTP protocol specification [6].

The common information model of the OpenTox domain objects is based on the Resource Description Framework (RDF) and described by the OpenTox ontology [8]. It should be noted that the initial design of the OpenTox API (version 1.0) was based on a XML schema, but it was later decided to adopt RDF as a more powerful approach to describe objects and their relationships, as well as to facilitate the reuse of ongoing ontology developments in bioinformatics. Any entity could be described in RDF as a collection of triples (or statements), each triple consisting of a subject, a predicate, and an object. The predicate (also called a property) denotes the relationship between two objects (e.g. *Model1 has_training_dataset Dataset1*). The objects are modelled in RDF as *Classes *(*rdf:Class*), and Classes have specific *Instances*. Relationships are modelled with *Properties *(*rdf:Property*).

Thus, the Resource Description Framework allows defining a data model (how the data is organized), instead of specifying data format (how the data is written into a file). Once a data model is defined, it could be serialized into different formats, for example RDF/XML [9], N3 [10], TURTLE [11]. The OWL Web Ontology Language [12] is built on top of RDF, and, compared to RDF, imposes restrictions on what is allowed to be represented. Because of such restrictions, the OWL subsets OWL-Lite and OWL-DL (Description Logic) allow performing automated machine reasoning. In OWL, there are Object properties and Data properties (*owl:Property*, which is a subclass of *rdf:Property*). An Object property specifies a relation between Instances, while a Data property specifies a relation between an Instance and a simple data value (string, integer, etc.). Properties cannot be used as Classes and vice versa.

Both REST and RDF technologies encourage data model development and consider assigning resource identifiers important. However, there are differences, as REST identifiers are used as addresses of the underlying protocol (e.g. HTTP URIs) and it is essential that URIs are dereferenceable. While the RDF representation allows HTTP URIs as resource identifiers, these are considered names, not addresses, and are not necessarily dereferenceable. HTTP URIs are hierarchical, while RDF does not exploit the hierarchy, and splits HTTP URIs into a prefix and identifier instead. REST resources define clear boundaries between information entities, while data, represented via RDF, is usually perceived as one linked graph. The common usage of RDF for data integration is to convert data coming from diverse sources into a (typically read only) single triple storage and provide a query interface (SPARQL endpoint). On the contrary, web services provide distributed and dynamically generated information. Most REST services define data formats [13] as a means for communication, rather than an explicit data model. The simultaneous use of RDF and REST is not yet widespread and there are ongoing debates on various related topics. Nevertheless, there is an added value of merging both technologies for independent deployments of multiple services, able to dynamically generate linked data with dereferenceable links. This could lead to an enrichment of the information space and scalability, in a manner similar to a deployment of many web servers that provide hypertext documents.

The OpenTox framework integrates both technologies into a distributed web services framework, where both data and processing resources are described by ontologies: either existing ones, or developed within the project. The framework consists of simple modules, developed by different partners and with different programming languages, running on a set of geographically dispersed servers, and communicating via Internet. The modules can be used to build more complex use cases, embed OpenTox web services into workflows, build web mashups, consume the web services via rich client applications, etc.

This paper describes a particular implementation of a subset of OpenTox web services, based on the AMBIT [14,15] project. AMBIT is an open source software for chemoinformatics data management, which consists of a database and functional modules, allowing a variety of queries and data mining of the information stored in a MySQL [16] database. The modules were initially designed and developed to serve as building blocks of a desktop application (AmbitXT), as per the requirements of a CEFIC LRI [17] contract. The AmbitXT application features a Swing graphical user interface, and provides a set of functionalities to facilitate the evaluation and registration of chemicals according to the REACH requirements: for example workflows for analogue identification and assessment of Persistence, Bioaccumulation, and Toxicity (PBT). The downloadable installer includes a large database, covering all REACH registered chemicals, as well as several publicly available datasets featuring toxicity data. Users can also import their own sets of chemical structures and data. Downloading and running the application locally on the user machine is usually considered an advantage, especially when handling confidential data. On the other hand, with the growing popularity of the Web browser as a platform for applications, cumbersome downloads of custom desktop solutions are becoming less convenient nowadays and are even considered obsolete sometimes.

The AMBIT software was considerably enhanced within the framework of the OpenTox project, not only by providing an OpenTox API compliant REST web service interface to most of its functionalities, but also by adding the ability to describe data, algorithms, and model resources via corresponding ontologies and to build QSAR models. AMBIT REST web services are distributed as web archive (*war *file) and can be deployed in an Apache Tomcat [18] application server or any other compatible servlet [19] container. All Toxtree [20,21] modules for predicting the toxicological hazard of chemical compounds are also integrated within this package and available as REST web services via the OpenTox model API. In addition, a separate project [22], implementing an OpenTox Ontology service, has been created. It consists of a simple implementation of a triple storage, exposing a SPARQL endpoint, and allowing RESTful updates via HTTP POST and DELETE commands.

## Implementation

AMBIT is implemented in Java, uses a MySQL database as backend, and relies on The Chemistry Development Kit [23-25] for cheminformatics functionality. The OpenTox API implementation introduces two additional major dependencies, namely, the Restlet [26] library for implementation of REST services, and the Jena [27] RDF API. Apache Maven [28] is used for software project management (organizing dependencies and building of executables). The source code is available in a Subversion repository at the SourceForge site [29]. There are two top level Maven projects, *ambit2-all *and *ambit2-apps*, consisting of several sub-modules. The first is used to organize and build modules, while *ambit2-apps *uses these modules as dependencies and builds the end user applications. The Toxtree source code [30] also includes dependencies on some of the *ambit-all *modules, and, on the other hand, is itself a dependency of the end user applications, in which it has been incorporated, such as AmbitXT and REST web services. The entire package currently consists of 30 Maven modules. The larger number of modules (30, compared to 21, as reported in the previous publication [15] that describes the standalone application), is mostly due to refactoring towards better organization of dependencies and introduction of new algorithms. The REST services implementation is organized in two modules, *ambit2-rest *and *ambit2-www*; the first one contains generic REST and RDF functionality, while the second is an implementation of the OpenTox API and builds the web application used to run AMBIT REST services.

Table 1 provides a non-exhaustive overview of the most important objects and operations of the OpenTox API, implemented by the AMBIT services. The complete description of the API [1] includes specifications of the input parameters and the result codes. An up-to-date version is available from the dedicated wiki at the OpenTox web site [31]. Currently, there is no AMBIT implementation of the OpenTox validation and reporting services; however, remote validation and reporting services are compatible, and can be used to validate models created via AMBIT services. Incorporation of the Authentication and Authorization API is under development.

**Table 1:** Summary of the OpenTox API, implemented by AMBIT REST services. {service} defaults to "ambit2".

1. Chemical compound http(s)://host:port/{service}/compound/{id}	
GET	Retrieves a representation of the chemical compound (in Chemical MIME [32] formats or image/* formats)

PUT	Updates the chemical compound

POST	Creates a new chemical compound, returns the URI of the new chemical compound

DELETE	Deletes the chemical compound

**2. Features (or properties) of chemical compounds http(s)://host:port/{service}/feature/{id}**	

GET	Retrieves a representation of the property (RDF representation of the *ot:Feature *class)

PUT	Updates the property representation

POST	Creates a new property, returns a URI of the new property

DELETE	Deletes the property

**3. Datasets of chemical compounds http(s)://host:port/{service}/dataset/{id}**	

GET	Retrieves a representation of the dataset (RDF representation of the *ot:Dataset *class, or Chemical MIME formats)

PUT	Updates the dataset (adds new compounds and/or properties)

POST	Creates a new dataset (e.g. uploads a file, which is transformed into a dataset). Returns the new dataset URI, or a Task URI for long running jobs. Parameters are expected in the body of the POST command, several formats are supported via the "Content-type" HTTP header, including RDF and chemical MIME formats.

DELETE	Deletes the dataset

**4. Algorithms http(s)://host:port/{service}/algorithm/{id}**	

GET	Retrieves a representation of the algorithm (RDF representation of the *ot:Algorithm *class)

POST	• Given a compound URI, or a dataset URI as input parameter, launches the processing algorithm and returns the URI of the dataset, containing the results, or returns a Task URI for long running jobs.
	• If the algorithm is a machine learning one, given a dataset URI as input parameter, builds a new predictive model and returns the Model URI, or the Task URI.
	• Parameters are expected in the body of the POST command, in "application/x-www-form-urlencoded" MIME format.
	• A common required parameter is dataset_uri = http://host:port/{service}/dataset/{datasetid}, which specifies the data set to be operated on.

**5. Models http(s)://host:port/{service}/model/{id}**	

GET	Retrieves a representation of the model (RDF representation of the *ot:Model *class)

POST	• Given a compound URI, or a dataset URI as input parameter, applies the predictive model, and returns the URI of the dataset, containing the predictions, or a Task URI for long running jobs.
	• Parameters are expected in the body of POST command, in "application/x- www-form-urlencoded" MIME format.
	• A common required parameter (in the body of the POST command is dataset_uri = http://host:port/{service}/dataset/{datasetid}, which specifies the data set to be operated on.

DELETE	Deletes the model

**6. Task http(s)://host:port/{service}/task/{id}**	

GET	Retrieves a representation of the task (RDF representation of the *ot:Task *class). The task status can be one of Queued, Running, Cancelled, Error or Completed.

DELETE	Cancels the task

**7.Query for compounds http(s)://host:port/{service}/query/compound/{identifier}/{results}**	

GET	Returns compounds, features and feature values, matching the submitted search criteria, in supported formats (dataset representation in RDF, SDF, MOL or SMILES format), according to the mime type requested by the corresponding Accept header:
	• *identifier *- any *name*, *formula*, *registry identifier*, *InChI *, *SMILES*, or predefined string "*url*"; if the string "*url*" is used, a query parameter is expected ?*search*=<*compound url*>
	• *results *- predefined strings: *'names'*, *'stdinchi'*, *'all'*, *'smiles'*, *'stdinchikey'*

**8. Ontology http(s)://host:port/ontology**	

GET	Submits a SPARQL query and retrieves the result in "application/sparql-results+xml" format. Requires an input parameter *query *= "SPARQL"

POST	Submits a SPARQL query and retrieves the result in "application/sparql-results+xml" format. Requires an input parameter *query *= "SPARQL" in the message body in "application/x-www-form- urlencoded" MIME format. Useful for submitting long queries that might exceed the supported URI length.

POST	Reads the RDF representation of an OpenTox object and adds it into the triple storage. Requires input parameter *uri=*"*OpenTox resource URI*"

DELETE	Deletes all RDF triples that refer to a given URI. Requires an input parameter *uri=*"*OpenTox resource URI*"

The RDF representation of OpenTox objects is defined by the OpenTox ontology. The current version is available at http://www.opentox.org/api/1.1/opentox.owl The namespace prefix used in this paper is "ot:", e.g. *ot:Model *refers to the http://www.opentox.org/api/1.1/opentox.owl#Modelclass. OpenTox REST resources are instances of the relevant RDF classes (e.g. http://apps.ideaconsult.net:8080/ambit2/model/9 is an instance of the *ot:Model *class). Appendixes 1 and 2 provide examples how to retrieve the representations of an OpenTox model and algorithm, respectively. As a consequence of being exposed as REST web services, all OpenTox objects URIs are dereferenceable. The examples provided in the Appendixes rely on the cURL [33] command line tool for transferring data with URI syntax, which supports all HTTP operations (as well as other protocols). Any tool or programming language library, supporting the HTTP protocol, can be used to communicate with the OpenTox REST services. The examples use live demo instances of the AMBIT implementation of the services, but are also applicable, with minor trivial changes, to any OpenTox compliant service.

### Appendix 1: An example how to retrieve the representation of an OpenTox model

curl -H "Accept:text/n3" http://apps.ideaconsult.net:8080/ambit2/model/9

@prefix ot:   <http://www.opentox.org/api/1.1#>.

@prefix dc:   <http://purl.org/dc/elements/1.1/>.

@prefix rdfs:   <http://www.w3.org/2000/01/rdf-schema#>.

<http://apps.ideaconsult.net:8080/ambit2/model/9>

   a   ot:Model ;

   dc:title "XLogP" ;

   ot:algorithm

<http://apps.ideaconsult.net:8080/ambit2/algorithm/org.openscience.cdk.qsar.descriptors.molecular.XLogPDescriptor>;

   ot:predictedVariables

      <http://apps.ideaconsult.net:8080/ambit2/feature/22114>.

<http://apps.ideaconsult.net:8080/ambit2/feature/22114>.

   a   ot:Feature.

<http://apps.ideaconsult.net:8080/ambit2/algorithm/org.openscience.cdk.qsar.descriptors.molecular.XLogPDescriptor>

   a   ot:Algorithm

### Appendix 2: An example how to retrieve the representation of an OpenTox algorithm

curl -H "Accept:text/n3" \

http://apps.ideaconsult.net:8080/ambit2/algorithm/org.openscience.cdk.qsar.descriptors.molecular.XLogPDescriptor

@prefix ot:   <http://www.opentox.org/api/1.1#>.

@prefix dc:   <http://purl.org/dc/elements/1.1/>.

@prefix rdfs:   <http://www.w3.org/2000/01/rdf-schema#>.

@prefix bo:   <http://www.blueobelisk.org/ontologies/chemoinformatics-algorithms/#>.

@prefix xsd:   <http://www.w3.org/2001/XMLSchema#>.

@prefix ota:   <http://www.opentox.org/algorithmTypes.owl#>.

<http://apps.ideaconsult.net:8080/ambit2/algorithm/org.openscience.cdk.qsar.descriptors.molecular.XLogPDescriptor>

   a   ot:Algorithm, ota:DescriptorCalculation ;

   dc:title "XLogP"^^xsd:string ;

   bo:instanceOf bo:xlogP.

The examples provided in Appendixes 3 and 4 illustrate how processing is performed via HTTP operations. The *dataset_uri *parameter refers to the ToxCast [34] dataset, which consists of 320 chemicals, and is essentially an example of batch processing via the OpenTox API.

### Appendix 3: An example of launching XLogP prediction for a dataset

curl -H "Accept:text/uri-list" -X POST -d "dataset_uri=http://apps.ideaconsult.net:8080/ambit2/dataset/112" \

http://apps.ideaconsult.net:8080/ambit2/model/9 -v

< HTTP/1.1 202 Accepted

http://apps.ideaconsult.net:8080/ambit2/task/232289a2-2ce8-4f2e-9a62-8db02887577b

Note that both the dataset and the models are accessed indirectly via URIs, so the only data transferred on input and output are those URIs, not actual content. The result is a Task URI, and the HTTP return code 202 Accepted is an indicator that the processing has not been completed yet. In case processing was completed, the return code would have been OK 200 and the returned URI - an *ot:Dataset*, where results could be retrieved.

### Appendix 4: An example of polling the status of asynchronous job (Task URI)

curl -i -H "Accept:text/uri-list" \

http://apps.ideaconsult.net:8080/ambit2/task/232289a2-2ce8-4f2e-9a62-8db02887577b

HTTP/1.1 200 OK

http://apps.ideaconsult.net:8080/ambit2/dataset/112?feature_uris[]=http%3A%2F%2Fapps.ideaconsult.net%3A8080 %2Fambit2%2Fmodel%2F9%2Fpredicted

Finally, we retrieve the prediction results from the URI shown in Appendix 4. The prediction results (Appendix 5) are represented as *ot:Dataset *(e.g. table with variable number of columns), which consists of data entries (*ot:DataEntry*) relating compounds (e.g. rows) to features (columns, *ot:Feature*). The table "cells" are represented as instances of the *ot:FeatureValue *class. A short excerpt, consisting of only two data entries (out of the total of 320 data entries included in this particular dataset), is shown in Appendix 5.

### Appendix 5: An example of prediction results retrieval by HTTP GET command on URI, received as shown in Appendix 4

curl -H "Accept:text/n3" \

"http://apps.ideaconsult.net:8080/ambit2/dataset/112?feature_uris%5B%5D=http%3A%2F%2Fapps.ideaconsult.net%3A8080%2Fambit2%2Fmodel%2F9%2Fpredicted"

@prefix ot:   <http://www.opentox.org/api/1.1#>.

@prefix dc:   <http://purl.org/dc/elements/1.1/>.

@prefix:   <http://apps.ideaconsult.net:8080/ambit2/>.

@prefix rdfs:   <http://www.w3.org/2000/01/rdf-schema#>.

@prefix owl:   <http://www.w3.org/2002/07/owl#>.

@prefix xsd:   <http://www.w3.org/2001/XMLSchema#>.

@prefix rdf:   <http://www.w3.org/1999/02/22-rdf-syntax-ns#>.

@prefix otee:   <http://www.opentox.org/echaEndpoints.owl#>.

[] a   ot:Dataset ;

   ot:dataEntry

      [a   ot:DataEntry ;

       ot:compound http://apps.ideaconsult.net:8080/ambit2/compound/147678/conformer/419677> ;

       ot:values

         [a   ot:FeatureValue ;

          ot:feature <http://apps.ideaconsult.net:8080/ambit2/feature/22114> ;

          ot:value "2.74"^^xsd:double

         ]

      ] ;

   ot:dataEntry

      [a   ot:DataEntry ;

       ot:compound <http://apps.ideaconsult.net:8080/ambit2/compound/2146/conformer/419678> ;

       ot:values

         [a   ot:FeatureValue ;

          ot:feature <http://apps.ideaconsult.net:8080/ambit2/feature/22114> ;

          ot:value "1.59"^^xsd:double

         ]

      ].

<http://apps.ideaconsult.net:8080/ambit2/algorithm/org.openscience.cdk.qsar.descriptors.molecular.XLogPDescriptor>

   a   ot:Algorithm.

<http://apps.ideaconsult.net:8080/ambit2/feature/22114>

   a   ot:Feature, ot:NumericFeature ;

   dc:title "XLogP" ;

   ot:hasSource

<http://apps.ideaconsult.net:8080/ambit2/algorithm/org.openscience.cdk.qsar.descriptors.molecular.XLogPDescriptor> ;

   =   otee:ENDPOINT_Octanol-water_partition_coefficient.

**The Ontology Service **is a separate project, which does not depend on AMBIT modules, and which compiles into a different web application. It currently uses the Jena TDB [35] persistence mechanism, and was initially designed as a proof-of-concept service to illustrate the added value of gathering RDF triples of several remote OpenTox services into the same triple storage and enabling SPARQL queries. According to our experience, the performance of the TDB storage, especially when embedded as a web service and being concurrently accessed by many users, is not optimal, and other available solutions are being evaluated. Currently, it is planned to use the ontology service as a registry of all deployed OpenTox services (both local and remote).

**AMBIT REST services **is a web application that includes all resources listed in Table 1 except the ontology service. All OpenTox objects are implemented as subclasses of *org.restlet.resource.ServerResource *[36], and reside in the *ambit-www *module, which compiles into a single web archive (*ambit2.war*). The Algorithm and Task resources are implemented as in-memory objects. Compounds, Features, Datasets, and Models rely on a MySQL database backend. The common architecture is as follows.

#### GET operations

The *ServerResource *receives input parameters and creates a query object, which encapsulates the database query. The query object could be as simple as the definition of a resource retrieval by its primary key or it could represent more complex queries like searching by several parameters, similarity search, or substructure search (SMARTS) pre-screening. The query object is processed by a generic "batch processing" class, which retrieves domain objects (e.g. compounds, features, datasets, dataset entries, or models) one by one, applies further processing if necessary, and serializes back from the server to the client the resource representation in the desired format by a "reporter" class. This setup allows for easy handling of new query types (by adding new query classes) and for adding many serialization formats (by writing new reporter classes). The supported MIME types for datasets (besides the mandatory *application/rdf+xml*) currently are: *chemical/x-mdl-sdfile, text/n3, application/x-turtle, chemical/x-mdl-molfile, chemical/x-cml, chemical/x-daylight-smiles, chemical/x-inchi, text/x-arff, application/pdf, text/uri-list, text/csv, text/plain*. Experimental support for YAML and JSON is also available. The most efficient implementation of a "reporter" class is to serialize the domain objects into the stream immediately after receiving them, without keeping the objects, or any related data, in memory. Unfortunately, when Jena is used to generate a RDF representation of a domain object, it requires building the entire RDF triple model prior to serialization. To avoid this overhead, the dataset RDF/XML serialization was re-implemented to use the Streaming API for XML (StAX) [37], resulting in reduced response time of dataset retrieval (2-10 times improvement, depending on the size of the dataset).

#### POST and PUT operations

Instances of *ServerResource *receive input parameters, create a task resource, put it into an execution queue, and immediately return the task URI and representation in the requested MIME type to the client. The execution queue consists of *java.util.concurrent.Ca llable* objects [38], while completed tasks are light objects, containing only input and output URIs. The result, as per the OpenTox REST API, is always a URI: either representing the result, or an intermediate Task object. The tasks are available via the Task service (Table 1), and are used, via GET, for accessing either the status of a unfinished task, or the URI of the results - for the completed ones. This defines a generic processing scheme where, for implementing new type of processing (e.g. integrating a new algorithm), it is sufficient to subclass the *ServerResource *and attach the specific type of *Callable *object that implements the new algorithm.

POST and PUT on datasets, compounds, and feature resources are used to create new resources or update the content of existing ones, and always return the URI of the new resources or the URI of the updated ones. POST on machine learning algorithms (e.g. regression, classification, or clustering) creates a new model resource and returns its URI. The representation of a model URI can be retrieved via GET to inspect the model details (e.g. training dataset, independent variables, specific parameters). POST on a model URI creates a new dataset, containing prediction results, and returns its URI. Returning the URI of a subordinate resource upon POST is in compliance with REST recommendations (and HTTP specifications [6]), as the content of the result URI could be later accessed via GET, obeying the cacheability constraint of the architecture. Neither REST nor HTTP strictly defines the meaning of "subordinate" resource; we however consider the OpenTox API interpretation compliant to the REST architecture, because in all of the cases, presented above, POST on a resource creates a new dependent resource, and is defined in a uniform manner. An important difference to remote procedure call (RPC) based architectures is that the client does not send the complete data to be processed; the processing service receives only the data URI, which it uses to retrieve the appropriate representation when it needs the data. The distinction between information resources and their representations, which is considered a key feature of REST, enables the processing resource to choose the most appropriate representation (i.e. no additional data conversion is necessary!) and keep track of the data provenance by simply referring to the data URI and its relevant metadata. This design also allows to dynamically generate predictive models, immediately making them available online, and maintaining in the underlying representation of linked resources all the information required to reproduce the model building process, which was one of the initial design goals of the OpenTox framework.

The results of applying the REST constraints to information processing elements, like data analysis algorithms, leads to a change in the way of thinking, modelling, implementing, and perceiving data processing. From a point of view of the REST architecture, a data processing algorithm is just another resource that retrieves data, given its identifier, and creates a resulting resource with another identifier. The difference between the data and processing elements vanishes.

#### DELETE operations

Usually implemented by deleting objects from the database backend, the integrity is managed via a standard relational database foreign keys mechanism. Integrity between local and remote objects is not addressed. If a local object refers to a remote OpenTox object, e.g. predictions stored as an AMBIT dataset by a remote model, and the remote model service becomes unreachable, this will not be reflected in any way. This is similar to the generic problem of broken hyperlinks on the Web and might be addressed in future by some suitable keep-alive or synchronization mechanism.

#### RDF input/output

Jena in-memory models are used to read incoming RDF data and to serialize domain objects into RDF formats. The lack of streaming RDF readers and writers is a major disadvantage for the use of RDF for data transfer. A possible workaround is to introduce a persistent RDF storage, but the performance gain has still to be evaluated. Another disadvantage of making domain objects available in RDF is the lack of support from most popular scripting languages, used to build web applications (e.g. JavaScript). As a workaround, JSON (Java Script Object Notation) [39] serialization of RDF is considered, and although many proposals and implementations exist, there is currently no standard for JSON serialization. Two of the existing JSON libraries have been evaluated, with the results not encouraging - the volume of the JSON representation is comparable to that of RDF/XML, and the same is true for the corresponding memory consumption. Possible workarounds are either to build client applications in programming languages with good RDF support or to provide alternative formats with efficient streaming support. Fortunately, the REST architecture natively supports multiple representations per resource, which allows using the most appropriate format for carrying out a particular task.

A clear advantage of the availability of RDF representations for the OpenTox objects, data, algorithms, and models is that it allows to combine easily the RDF representations of remote resources into a standard triple storage, annotating and cross-linking objects with terms from existing ontologies. Publishing a dataset of chemical structures and their properties as linked data becomes as straightforward, as uploading a *sdf *file into an OpenTox dataset service, with optional subsequent annotation of property tags.

## Results and Discussion

We have implemented a large subset of the OpenTox API in the open source AMBIT REST package, and have made it available both as live demo online services and as a downloadable package, allowing third parties to install and run separate instances of the services, either on Intranet or publicly on the Internet.

The major advantage is the ability of the framework to hide implementation details and offer diverse functionality via a uniform application programming interface, which, while generic, allows encapsulating very diverse data and predictive algorithms and allows seamless integration of remote services. Additionally, representing domain objects via the Resource Description Framework allows to explicitly assert relationships between data and data generation processes.

### Uniform access to data

The OpenTox compound and dataset API provide generic means to access chemical compounds and aggregate various data. **Chemical compounds **are assigned unique URIs, and can be retrieved, created, or deleted via HTTP POST, PUT and DELETE commands, submitted to the compound service *http://host:port/{service}/compound*. The GET command returns a representation of the chemical compound in a specified MIME format (Appendix 6). Changing the MIME format in this example will return the representation of the compound in that format, making the service essentially work as a format converter.

### Appendix 6: An example of retrieving a compound in a specified format (Chemical MIME for SMILES in this example)

curl -H "Accept:chemical/x-daylight-smiles" http://apps.ideaconsult.net:8080/ambit2/compound/1

O=C

The concept of a **dataset of chemical compounds **is central to the OpenTox web services functionality. Algorithm services accept a dataset URI in order to build a model or to generate descriptor values. Model services accept a dataset URI in order to apply a model and obtain predictions. Predictions are also returned as a dataset URI, whose contents could be subsequently retrieved (Appendix 5). Search results (by identifiers, similarity, or substructure), are available as datasets as well.

The OpenTox Dataset (*ot:Dataset *class) can be thought of as a file of chemical compounds, along with their properties, which is identified (and referred to) by a unique web address, instead of a filename, and can be read and written remotely. The dataset POST operation allows uploading datasets in RDF representation, as well as files with chemical structures with arbitrary set of fields. AMBIT services do not restrict entering and uploading data to predefined fields only. Instead, arbitrary data can be imported, and later annotated to establish the semantics of the fields. When uploading data in RDF format, the client has full control of the fields' representation. This is a substantial improvement over most of the current practices with popular chemical formats, which usually involve describing the meaning of the fields in separate documents, targeted at human readers; sadly, this approach tends to lead to quite frequent peculiarities.

### Appendix 7: A RDF representation of a single entry from the DSSTox Carcinogenic Potency Database dataset

@prefix ot:   <http://www.opentox.org/api/1.1#>.

@prefix dc:   <http://purl.org/dc/elements/1.1/>.

@prefix:   <http://apps.ideaconsult.net:8080/ambit2/>.

@prefix otee:   <http://www.opentox.org/echaEndpoints.owl#>.

@prefix rdfs:   <http://www.w3.org/2000/01/rdf-schema#>.

@prefix owl:   <http://www.w3.org/2002/07/owl#>.

@prefix xsd:   <http://www.w3.org/2001/XMLSchema#>.

@prefix ac:   <http://apps.ideaconsult.net:8080/ambit2/compound/>.

@prefix ad:   <http://apps.ideaconsult.net:8080/ambit2/dataset/>.

@prefix rdf:   <http://www.w3.org/1999/02/22-rdf-syntax-ns#>.

@prefix af:   <http://apps.ideaconsult.net:8080/ambit2/feature/>.

af:21611

   a   ot:Feature ;

   dc:title "ActivityOutcome_CPDBAS_Mutagenicity" ;

   ot:hasSource ad:10 ;

   =   otee:Mutagenicity.

af:21604

   a   ot:Feature ;

   dc:title "TD50_Dog_mg" ;

   ot:hasSource ad:10 ;

   ot:units "mg" ;

   =   otee:ENDPOINT_Carcinogenicity.

ac:144089

   a   ot:Compound.

ad:10

   a   ot:Dataset ;

   ot:dataEntry

      [ a   ot:DataEntry ;

       ot:compound ac:144089 ;

       ot:values

         [a   ot:FeatureValue ;

          ot:feature af:21604 ;

          ot:value "blank"^^xsd:string

         ] ;

       ot:values

         [a   ot:FeatureValue ;

          ot:feature af:21611 ;

          ot:value "active"^^xsd:string

         ]

      ].

A simple example is representing carcinogenicity data from two public datasets, DSSTox CPDBAS: Carcinogenic Potency Database [40] (Appendix 7) and ISSCAN: Chemical Carcinogens Database [41]. Both datasets are available as *sdf *files, with fields described in human readable documents. The outcome of the carcinogenicity study is represented in the "*ActivityOutcome*" field in CPDBAS (with allowed values *"active", "unspecified", "inactive*"), while in ISSCAN, a numeric field named "*Canc*" is used with allowed value of 1, 2, or 3. The description of the numbers (*3 = carcinogen; 2 = equivocal; 1 = noncarcinogen) *is only available in a separate "Guidance for Use" *pdf *file. Ideally, toxicity prediction software should offer comparison between the data and models, derived from both datasets, which is currently impossible without involving human efforts to read the guides and establish the semantic correspondence between the relevant data entries if and when possible. Moreover, every toxicity prediction package has to do the same. The two files in the example are selected only because they are publicly available and widely known. This is a typical scenario of the current state of representing toxicity data. Even if the toxicity data is highly structured within a commercial or in-house database, the usual practice for exchanging it is through export into unstructured file formats. ToxML [42] is a notable example of an attempt of a structured file format for data exchange in toxicology, but it has not yet been adopted beyond its original authors, even though there are ongoing efforts in this direction [43]. There are a variety of relevant ontology development efforts [44,45], but these are in most cases done in a different context, and are only partially applicable to representations of toxicology studies.

Being aware of the lack of standards in this area, the authors of the OpenTox API have designed it in a way to provide a generic approach towards data representation, keeping the flexibility of importing arbitrary named fields, but still allowing assignment of computer readable annotations to the fields. This is illustrated in Appendixes 8 and 9.

### Appendix 8: A RDF representation of the "Canc" field of the ISSCAN dataset, available via AMBIT services and OpenTox API (prefixes are the same as in Appendix 7, and therefore omitted)

ad:9 a ot:Dataset ;

   rdfs:seeAlso "http://www.epa.gov/NCCT/dsstox/sdf_isscan_external.html" ;

   dc:source "ISSCAN_v3a_1153_19Sept08.1222179139.sdf" ;

   dc:title "ISSCAN: Istituto Superiore di Sanita, CHEMICAL CARCINOGENS: STRUCTURES AND EXPERIMENTAL DATA".

af:21573

   a ot:Feature ;

   dc:title "Canc" ;

   ot:hasSource ad:9 ;

   =   otee:ENDPOINT_Carcinogenicity.

The fields in *sdf *files and other formats can contain arbitrary attributes, which are represented as instances of the *ot:Feature *class from the OpenTox ontology. Every feature is identified by a unique URI, which is hosted at a feature service (*http://host:port/{service}/feature*) and is dereferenceable (a representation of the feature can be retrieved through a GET command). The RDF representation includes a feature name (via *dc:title *property), units (via *ot:units *property), and a link to the resource (via *ot:hasSource*) that was used to generate this property or where it was originally read from. Currently, the range of *ot:hasSource *property is defined to be one of *ot:Algorithm*, *ot:Model*, or *ot:Dataset*. Using the *owl:sameAs *property, it is possible to assert that an instance of the *ot:Feature *class is the same as another resource, defined in some other ontology. An example is shown in Appendix 8, where the feature *af:21573 *is asserted to be the same as the otee:*ENDPOINT_Carcinogenicity *individual from a simple ontology [1] that enables the representation of physicochemical properties and toxicology endpoints as defined in the ECHA guidance document [46]. The same approach, as well as using the *rdf:type *property, can be applied to assign more elaborate representations of toxicity studies to a particular feature, provided that an ontology describing the study exists. This technique is used to represent the ToxCast data in AMBIT services, and enables linking and querying related entries from the GO ontology [47].

### Appendix 9: A RDF representation of a subset of fields of the CPDBAS dataset, available via AMBIT services and OpenTox API (prefixes are the same as in Appendix 7, and therefore omitted)

af:21603

   a   ot:Feature ;

   dc:title "STRUCTURE_MolecularWeight" ;

   ot:hasSource ad:10 ;

   =   <http://example.org#an-ontology-entry-representing-molecular-weight>.

af:21607

   a   ot:Feature ;

   dc:title "STRUCTURE_ChemicalName_IUPAC" ;

   ot:hasSource ad:10 ;

   =   <http://example.org#an-ontology-entry-representing-IUPAC name>.

af:21610

   a   ot:Feature ;

   dc:title "ActivityOutcome_CPDBAS_Rat" ;

   ot:hasSource ad:10 ;

   =   otee:ENDPOINT_Carcinogenicity.

ad:10

   a   ot:Dataset ;

   rdfs:seeAlso "http://www.epa.gov/NCCT/dsstox/sdf_cpdbas.html" ;

   dc:title "CPDBAS: Carcinogenic Potency Database Summary Tables - All Species".

Instances of the *ot:Feature *class (Appendix 9) are used to represent arbitrary properties, including chemical identifiers (e.g. *STRUCTURE_ChemicalName_IUPAC*), properties like molecular weight (e.g. *STRUCTURE_MolecularWeight*), or calculated descriptors (Appendix 5) and model predictions (Appendix 11). If *ot:hasSource *points to an OpenTox algorithm or model URI, it could be directly used to launch the calculations for any new compound or dataset by simply initiating a HTTP POST to this URI, with an input parameter, pointing to the compound or dataset. This ensures keeping track of all the processing steps performed by the OpenTox services, and provides sufficient information to reproduce or repeat the calculations (Appendix 5). Features can be deleted by sending a DELETE command to the feature service, and created or updated via POST and PUT commands by providing a RDF representation as an input parameter. AMBIT services automatically create features when a dataset is being uploaded. If the uploaded dataset is not in RDF format, the features are generated with *dc:title *equal to the field name in the file and *ot:hasSource *property linking to the dataset, the combination of both properties used as a unique key. The features representation can be modified and annotated later by sending an appropriate RDF representation to the feature URI via a HTTP PUT command.

The use of dynamically generated and dereferenceable URIs for RDF resource identifiers differs from the classic recommendation of using "stable" identifiers from a predefined ontology. However, we consider the dynamically generated RDF graph an advantage of OpenTox services, and, moreover, it does not preclude linking dynamically generated resources with equivalent resources that have stable identifiers, if such exist. For example, features are expected to be associated via *owl:sameAs *links with stable identifiers, describing specific chemical properties. Arbitrary RDF statements, including both dynamically generated and stable resources could be added as well. The dynamically generated RDF representations allow quickly publishing information in RDF format and making it available online. Models and predictions also immediately become available as RDF resources online, and include live local and remote links, keeping track of the provenance (how predictions have been calculated and where the data came from). Given the availability of the OpenTox services as open source, anybody interested could run an instance of the services themselves, for as long as necessary. Because of the interoperable and distributed design, multiple instances of services running at multiple places could communicate and generate dynamically linked data. The URIs and addresses of networking resources generally don't have infinite lifetime, but this is not considered disadvantage for the World Wide Web, where, if any piece of the dynamic infrastructure is perceived important - for economic or any other reasons - it will certainly remain available for longer than average. The fact that HTTP URIs are transient and dependent on the service location is a consequence of the early Internet design as a medium for host-to-host communication, rather than one for data access, and also of the lack of location independent application names in Internet protocols [48]. Revising the current status of network resources naming towards persistent and self-certifying names and content-oriented networking is a field of active research nowadays, and may render the disputes about dereferenceability and stability of resource identifiers irrelevant in future.

Finally, it is trivial to retrieve the RDF representations from an arbitrary set of geographically distributed services. It is equally easy to create a snapshot of the content of a given subset of services of particular interest, either for archiving purposes, or in order to import it into a RDF triple storage and expose it via a SPARQL endpoint.

We support the view [49,50] that the current practice of aggregating data via loading RDF dumps into a single triple store is not always the best approach, but rather a temporary solution, until emerging technologies for distributed querying and reasoning become more efficient and scalable enough to eliminate the need of centralized data stores. Meanwhile, web services as the OpenTox REST ones, that provide dynamically generated RDF data via resolvable identifiers, can be crawled in a similar way as search engines crawl the web. However, there is the additional benefit of results being retrieved and reasoning performed over structured data, instead of just analysing keywords and links as popular search engines typically operate today.

### Uniform approach to data processing, model building, and predictions

The ability to represent data in a generic way, as explained above, greatly simplifies **data processing**. The latter can be described as the following three simple steps:

1. Read data from a web address, representing an *ot:Compound *or an *ot:Dataset *instance;

2. Perform processing; store results as *ot:Dataset *representation (e.g. *ot:FeatureValue *instances);

3. Write the *ot:Dataset *RDF representation to an OpenTox data service; return the URI of the resulting dataset.

The OpenTox API specifies two classes that perform processing - *ot:Algorithm *and *ot:Model*, supported by *http://host:port/{service}/algorithm *and *http://host:port/{service}/model *services, respectively. The lists of available algorithms can be retrieved by a GET command. The type of the algorithm is specified by sub-classing the algorithm instance from the respective class in the Algorithm types ontology [1]. Two major types of algorithms are data processing ones and model building algorithms.

Models are generated by the respective algorithms, given specific parameters and data. The process of **model creation **(e.g. using statistical algorithm to build a model) is initiated by sending a POST command to the algorithm service (example available in the Supporting Information [Additional file 1]), and involves the following steps:

1. Optionally read data from a web address, representing an *ot:Dataset *instance;

2. Create a model; describe it as an *ot:Model *instance; this includes specifying *ot:Feature *instances that contain the results, via the *ot:predictedVariables *property, as well as linking any independent and target variables via the *ot:independentVariables *and the *ot:dependent *variables properties;

3. Assign a unique URI to the model, and return the URI;

4. A POST command to the model URI, with a dataset or compound URI as input parameter, could be later used to calculate predictions.

This architecture turns out to be successful in encapsulating different algorithms and models in a single API. A summary of the algorithms, included in AMBIT REST services, is shown in Table 2 and the full list can be retrieved originally from http://apps.ideaconsult.net:8080/ambit2/algorithm or from http://host:port/ambit2/algorithm in any other installation of the *ambit2.war*.

**Table 2:** Algorithms, implemented in AMBIT REST services

CDK descriptors	http://host:port/ambit2/algorithm/org.openscience.cdk.qsar.descriptors.molecular.*
Weka [51] machine learning algorithms	http://host:port/ambit2/algorithm/LR
	http://host:port/ambit2/algorithm/SimpleKMeans
	http://host:port/ambit2/algorithm/J48

Toxtree [20] modules	http://host:port/ambit2/algorithm/toxtreecarc
	http://host:port/ambit2/algorithm/toxtreecramer
	http://host:port/ambit2/algorithm/toxtreecramer2
	http://host:port/ambit2/algorithm/toxtreeeye
	http://host:port/ambit2/algorithm/toxtreemichaelacceptors
	http://host:port/ambit2/algorithm/toxtreeskinirritation
	http://host:port/ambit2/algorithm/toxtreeskinsens
	http://host:port/ambit2/algorithm/toxtreemic
	http://host:port/ambit2/algorithm/toxtreeverhaar
	http://host:port/ambit2/algorithm/toxtreesmartcyp[52]

pKa [53] prediction	http://host:port/ambit2/algorithm/pka

Applicability domain algorithms [54-56]	http://host:port/ambit2/algorithm/distanceCityBlock
	http://host:port/ambit2/algorithm/distanceEuclidean
	http://host:port/ambit2/algorithm/fpmissingfragments
	http://host:port/ambit2/algorithm/fptanimoto
	http://host:port/ambit2/algorithm/leverage
	http://host:port/ambit2/algorithm/distanceMahalanobis
	http://host:port/ambit2/algorithm/nparamdensity
	http://host:port/ambit2/algorithm/pcaRanges

Superservice	http://host:port/ambit2/algorithm/superservice

InChI	http://host:port/ambit2/algorithm/ambit2.descriptors.InChI??

MOPAC descriptors	http://host:port/ambit2/algorithm/ambit2.mopac.
	*MopacOriginalStructure*

3D structure optimization via MOPAC	http://host:port/ambit2/algorithm/ambit2.mopac.MopacShell

SOME [57]	http://host:port/ambit2/algorithm/ambit2.some.
	*DescriptorSOMEShell*

MCSS	http://host:port/ambit2/algorithm/mcss

Fingerprints, used in similarity searching	http://host:port/ambit2/algorithm/fingerprints

Generate data for accelerating substructure search	http://host:port/ambit2/algorithm/smartsprop
	http://host:port/ambit2/algorithm/struckeys

Retrieve structures from non-OpenTox online services	http://host:port/ambit2/algorithm/finder

Most of the algorithms (except Weka and Toxtree) are considered data processing algorithms, and accept a dataset URI as input parameter, returning URI of the resulting dataset. The calculated values are included as feature values, as explained above. The structure optimization algorithm returns a dataset with links to the new 3D structures. SMARTCyp and SOME algorithms return their results as features as well, but the features represent calculated atomic properties. The MCSS algorithm accepts a dataset and creates a model, containing a set of maximum common substructures. The model could be further applied to new datasets or compounds. The superservice is an algorithm, which encapsulates descriptor calculation and model prediction, by automatically identifying which descriptors are required by a given model, launching the calculation, and, when results are available, applying the model itself. Toxtree algorithms are implemented as a model building algorithm, although being fixed rules and not requiring a training dataset. Thus, upon installation of the web application, the Toxtree model needs to be created by sending a HTTP POST to the corresponding algorithm. The Weka algorithms are selected to be representative of regression, classification, and clustering algorithms. They accept a dataset URI and a feature URI (referring to the target variable), and generate a model URI, as specified in the API. The implementation of Weka algorithms as OpenTox REST services is a generic one; inclusion of all algorithms, available in the Weka package, is just a matter of configuration, and the list will be extended in future releases. The RDF representation of all algorithms and models can be retrieved by submitting a GET command.

### Registering data, algorithms and models; SPARQL query

The OpenTox ontology service provides a place for registering OpenTox resources, running at remote places, as well as searching capabilities via SPARQL. Registering a resource into the ontology service requires sending a POST command to the service, with a parameter, pointing to the resource being registered (see Supporting Information [Additional file 1]). This allows a model, created by a remote service, to become available to any application that can send relevant queries to the ontology service. The registered resources and their properties could be retrieved via the service SPARQL endpoint (Appendix 10). Adding query conditions may restrict the search to models of specific type (e.g. regression) or toxicology endpoint of interest (e.g. carcinogenicity).

### Appendix 10: An example of retrieving information about a specific model (X and Y variables; learning algorithm; variables, containing the predictions; endpoints)

PREFIX ot:   <http://www.opentox.org/api/1.1#>

PREFIX ota:   <http://www.opentox.org/algorithms.owl#>

PREFIX owl:   <http://www.w3.org/2002/07/owl#>

PREFIX dc:   <http://purl.org/dc/elements/1.1/>

PREFIX rdfs:   <http://www.w3.org/2000/01/rdf-schema#>

PREFIX rdf:   <http://www.w3.org/1999/02/22-rdf-syntax-ns#>

PREFIX otee:   <http://www.opentox.org/echaEndpoints.owl#>

   SELECT ?Model ?algorithm ?xvars ?descriptorAlgorithms ?yvars ?endpoints ?predicted

      WHERE {

      ?Model rdf:type ot:Model.

   OPTIONAL {?Model dc:title ?title }.

   OPTIONAL {

      ?Model ot:algorithm ?algorithm.

      ?algorithm rdf:type <http://www.opentox.org/algorithmTypes.owl/#Regression>.

   }.

   OPTIONAL {

      ?Model ot:independentVariables ?xvars.

      OPTIONAL {?xvars ot:hasSource ?descriptorAlgorithms. }.

   }.

   OPTIONAL {

      ?Model ot:dependentVariables ?yvars.

      OPTIONAL {?yvars owl:sameAs ?endpoints. }.

   }.

   OPTIONAL {

      ?Model ot:predictedVariables ?predicted.

      OPTIONAL {?predictions owl:sameAs ?endpoints. }.

   }.

}

Any number of ontology services can be installed, thus allowing clustering and querying resources of interest to specific applications. Policies and access rights for protecting the resources are currently under development. Alternatively, a RDF triple storage of choice could be used to aggregate resources, generated by different implementations of OpenTox services.

A RDF graph, describing two models (*tumm:TUMOpenToxModel_kNN_92 *and *am:33*), running on remote services and using the same training dataset (*ot:trainingDataset ad:R545*) and descriptors (*ot:independentVariables af:22213, af:22137, af:22252, af:22127*; the link to the descriptor calculation service shown only for the *af:22127*), hosted and calculated by AMBIT services, is provided in Appendix 11.

### Appendix 11: A RDF graph, representing two remote models, using the same training dataset (the RDF content was aggregated by retrieving the RDF representations of multiple web services, and is available as Supporting Information [Additional file 2])

@prefix:   <http://apps.ideaconsult.net:8080/ambit2/>.

@prefix ot:   <http://www.opentox.org/api/1.1#>.

@prefix dc:   <http://purl.org/dc/elements/1.1/>.

@prefix tuma:   <http://opentox.informatik.tu-muenchen.de:8080/OpenTox-dev/algorithm/>.

@prefix tumm:   <http://opentox.informatik.tu-muenchen.de:8080/OpenTox-dev/model/>.

@prefix ota:   <http://www.opentox.org/algorithmTypes.owl#>.

@prefix otee:   <http://www.opentox.org/echaEndpoints.owl#>.

@prefix bo:   <http://www.blueobelisk.org/ontologies/chemoinformatics-algorithms/#>.

@prefix rdfs:   <http://www.w3.org/2000/01/rdf-schema#>.

@prefix am:   <http://apps.ideaconsult.net:8080/ambit2/model/>.

@prefix owl:   <http://www.w3.org/2002/07/owl#>.

@prefix xsd:   <http://www.w3.org/2001/XMLSchema#>.

@prefix ac:   <http://apps.ideaconsult.net:8080/ambit2/compound/>.

@prefix rdf:   <http://www.w3.org/1999/02/22-rdf-syntax-ns#>.

@prefix ad:   <http://apps.ideaconsult.net:8080/ambit2/dataset/>.

@prefix ag:   <http://apps.ideaconsult.net:8080/ambit2/algorithm/>.

@prefix af:   <http://apps.ideaconsult.net:8080/ambit2/feature/>.

tumm:TUMOpenToxModel_kNN_92

   a   ot:Model ;

   dc:title "OpenTox model created with TUM's kNNregression model learning web service." ; ot:algorithm tuma:kNNregression ;

   ot:dependentVariables

      af:22200 ;

   ot:independentVariables

      af:22213, af:22137, af:22252, af:22127 ;

   ot:predictedVariables

      af:27501 ;

   ot:trainingDataset ad:R545.

am:33

   a   ot:Model ;

   dc:title "Caco-2 Cell Permeability" ;

   ot:algorithm ag:LR ;

   ot:dependentVariables

      af:22200 ;

   ot:independentVariables

      af:22213, af:22137, af:22252, af:22127 ;

   ot:predictedVariables

      af:26182 ;

   ot:trainingDataset ad:R545.

ag:LR

   a   ot:Algorithm, ota:Supervised, ota:EagerLearning, ota:SingleTarget, ota:Regression;

   dc:title "Linear regression"^^xsd:string.

af:22127

   a   ot:Feature ;

   dc:title "FPSA-2" ;

   ot:hasSource

<http://apps.ideaconsult.net:8080/ambit2/algorithm/org.openscience.cdk.qsar.descriptors.molecular.CPSADescriptor >.

### Linked resources

**Figure 1:**
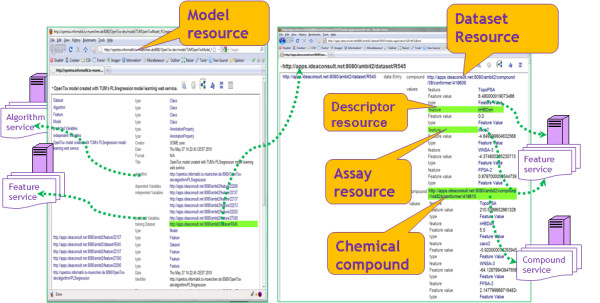
**Illustration of linked resources, generated by OpenTox services**.

Uploading data and running calculations via the OpenTox API and its implementation by AMBIT services generates a multitude of linked resources, all available via their RDF representations. The links could span many remote sites, running various implementations of OpenTox services. For example, a model, built by model services running at site A, will be accessible via its web address, but the representation could include links to the training dataset and prediction variables, hosted at OpenTox services running at site B. The features, representing predicted variables, contain links back to the remote model. An illustration of linked resources, generated by OpenTox services, is provided on Figure 1 and Additional file 2.

### Comparison with similar systems

The design of the OpenTox REST API and its implementation started at the beginning of the OpenTox FP7 project in 2008. At that moment we were not aware of any API with comparable functionality and design. There were examples of REST services in cheminformatics, but usually designed as a monolithic system and not available for download and installation elsewhere. The OpenTox framework is designed and developed collaboratively with the intention to be a modular and interoperable distributed system. The SADI framework [58,59] is the only other existing system which combines REST and RDF technologies to perform bio- and cheminformatics tasks. It should be noted, though, that these systems have been developed independently, without mutual awareness, until quite recently. While both approaches might seem similar to some extent, there are significant differences in their design and implementation.

The main goal of the OpenTox framework is to provide distributed means for building, using, and validating predictive models. We are not fully aware whether SADI services support generating and validating new predictive models via machine learning techniques or other methods. OpenTox services are independent, and can be mashed up or invoked in serial or parallel fashion by explicit invocation of command tools, existing workflow systems, or custom user interface. SADI's strong point is in the usage of implicit invocation of web services, given a SPARQL query. The SHARE engine [60] decides which services to invoke in order to fill in the missing data. The SADI services use HTTP, but define HTTP resources only for the processing elements, not for the data elements. The calculations are initiated by a POST command, and the data is returned in the body, resembling a typical processing by a remote procedure call, rather than a REST resource. Input data is subsumed into the output data, and neither of the data has its own dereferenceable identifier. OpenTox services work by accepting a URI of an input resource and return a URI of the resulting resource. The content of the latter could be retrieved by a subsequent GET operation if necessary - as a whole or in parts. This allows processing of datasets of arbitrary number of entries. Dataset is a central type of resource in OpenTox, while we are not aware of a corresponding concept in SADI. Implementation-wise, SADI services require a RDF triple storage as a backend, while OpenTox services do not mandate any particular backend representation; it is sufficient only to serialize resources to RDF on input/output in order to be compatible with the OpenTox API. Another difference exists due to the requirement to define a custom input/output format for each SADI processing service, while OpenTox services have a uniform interface, which resembles conceptually the standard input and output streams in UNIX operating systems, and brings proven flexibility when composing services into arbitrary workflows. Finally, SADI strives to provide a single ontology, describing all cheminformatics services. We believe that this is hardly achievable in a truly distributed system, and therefore designed OpenTox in a different way; we provide a skeleton ontology, allowing representation of a few basic classes, generate dynamic resources, and link/annotate them with all relevant third party ontologies.

### Applications

Although all AMBIT REST services support HTML MIME format and could be accessed through a web browser, the intended use is via custom client applications, which would consume the web services, and provide a friendly user interface, tailored to specific use cases. An example is the ToxPredict[1,61] web application, which provides a customized user interface for searching data, selecting and applying models, and displaying prediction results. Integration of REST services into workflow systems and rich client applications are other options, subject to further work.

### Installation

• Download the web application archive (*war*) file from http://ambit.sourceforge.net/

• Deploy the *war *file into a servlet container

• Ensure MySQL is installed and running at the default port

• Create an empty database by issuing a POST request to *http://host:8080/ambit2/admin/database *URI as shown in the command below. Note: *mysqlprivuser *should be an existing MySQL user with sufficient privileges to create a database.

curl -X POST -d "dbname = ambit2" -d "user = mysqlprivuser" -d "pass = mysqlprivpass" \ http://host:8080/ambit2/admin/database

• On success, reading the URI *http://host:8080/ambit2/admin/database *will return the database name

• Import your own data by sending a POST command to *http://host:8080/ambit2/dataset *or using the web interface. Use the OpenTox API to run algorithms and models.

**Plans for future developments **include protecting resources via the OpenTox Authentication and Authorization API [62], which relies on a customized OpenAM [63] service; extend dataset and feature representations to accommodate hierarchical data; develop applications with specialized user interfaces that would consume the services; improve and refactor the services' implementation in order to provide a skeleton code for easy deployment of third party algorithms and models, compliant with the OpenTox API; provide a client library for accessing the OpenTox API.

## Conclusions

The AMBIT REST services package has been developed as an extension of AMBIT modules, wrapping their functionalities as REST web services, and adding some new ones. This implementation covers a large subset of the functionality, specified by the OpenTox API, and is available both as live demo online web services and as a downloadable web application, which can be deployed in a compatible servlet container. The services, implementing the OpenTox API for compounds, datasets, and features, enable importing arbitrary files with chemical structures and their properties, allowing linking to computer readable information about the data fields, as well as keeping provenance information. In addition, they support multiple structures of the same compound, which is useful for storing and working with multiple conformations, as well as for comparing structures, originally residing in different source databases. Uploading a file with chemical structures and data makes it automatically available in several formats, including the mandatory RDF representation, defined by the OpenTox ontology. The services, implementing the OpenTox API for algorithms and models, provide a unified web service interface to several descriptor calculation, machine learning, and similarity searching algorithms, as well as to applicability domain and toxicity prediction models. The complexity and diversity of the processing is reduced to the simple paradigm "read data from a web address, perform processing, write to a web address". The online service allows running predictions without installing any software, as well sharing datasets and models between online users. The downloadable web application allows researchers to set up their own systems of chemical compounds, calculated and experimental data, and to run existing algorithms and create new models. The advantage of exposing the functionality via the OpenTox API is that all these resources could interoperate seamlessly, not only within a single web application, but also in a network of many cooperating distributed services.

Exposing functionalities through a web application programming interface allows to hide the implementation details of both data storage (different database types vs. memory vs. file system backend) and processing (descriptor calculation algorithms using CDK, OpenBabel, commercial or in-house implementations). The availability of data and processing resources as RDF facilitates integrating the resources as Linked Data [64]. The distributed algorithm and model resources automatically generate RDF representations, making the linked data dynamic, and not relying on a single traditional triple storage. The classes in the OpenTox ontology are designed to cover the minimum number of building blocks, necessary to create predictive toxicology applications. The OpenTox ontology relies on external ontologies to represent descriptor calculation algorithms, machine learning methods, and toxicity studies. We believe that such modularity better reflects how any particular domain is described in reality [65], compared to monolithic ontologies, which could be difficult or even impossible to reach consensus on, and would be hard to maintain. RDF natively provides means to link multiple concepts to a same resource, either by multiple inheritance, or *owl:sameAs *links, and we intend to use these techniques, together with the current dataset representation, to describe complex toxicological studies.

The AMBIT REST services package is one of the several independent implementations of the OpenTox Application Programming Interface, being developed within the OpenTox project. While creating an ontology (even for a rather limited domain) by consensus is a challenge by itself, the value of having multiple independent implementations of services using the ontology is enormous, as it clearly highlights components that have not been explicitly described, and are thus open to diverging and possibly conflicting interpretations. This demonstrates that the OpenTox API could be implemented equally well either as a completely new project or as an extension of an existing software. It also demonstrates OpenTox API's ability to provide a unified interface to diverse algorithms and data, and to encourage defining explicit relationships between data and processing routines. Last but not least, the services provide a sound basis for building web mashups, end user applications with friendly GUIs, as well as embedding the functionalities in existing workflow systems.

## Availability and requirements

• **Project name**: AMBIT implementation of OpenTox REST web services

• **Project home page**: http://ambit.sourceforge.net/

• **Operating system(s)**: Platform independent

• **Programming language**: Java

• **Other requirements**: Java 1.6 or higher, Tomcat 6.0 or higher, MySQL 5.1 or higher

• **License**: GNU LGPL (ambit2-all modules), GNU GPL (web services)

• **Any restrictions to use by non-academics**: None

• **Online web services**: http://apps.ideaconsult.net:8080/ambit2/

## Abbreviations

API: Application Programming Interface; CDK: The Chemistry Development Kit; HTTP: Hypertext Transfer Protocol; MIME: Multipurpose Internet Mail Extensions: (Q)SAR: (Quantitative) Structure Activity Relationship; REST: REpresentational State Transfer; RDF: Resource Description Framework; URI: Universal Resource Identifier.

## Competing interests

The authors declare that they have no competing interests.

## Authors' contributions

NJ performed most of the AMBIT web services software development, coordinated the OpenTox framework implementation, and provided valuable insights on a range of important aspects of the manuscript. VJ contributed to the design and testing of the webservices, performed data gathering and curation, and helped drafting the manuscript. All authors read and approved the final manuscript.

## Authors' information

Nina Jeliazkova (NJ): Nina received a M.Sc. in Computer Science from the Institute for Fine Mechanics and Optics, St. Petersburg, Russia in 1991, followed by a PhD in Computer Science (thesis "Novel computer methods for molecular modelling") in 2001 in Sofia, Bulgaria, and a PostDoc at the Central Product Safety department, Procter & Gamble, Brussels, Belgium (2002 - 2003). Her professional career started as a software developer first at the oil refinery Neftochim at Burgas, Bulgaria (1991 - 1995), then at the Laboratory for Mathematical Chemistry, Burgas, Bulgaria (1996 - 2001). She joined the Bulgarian Academy of Sciences in 1996 as a researcher and network engineer at the Network Operating Centre of the Bulgarian National Research and Education Network. She is founder and co-owner of Ideaconsult Ltd, and is technical manager of the company since 2009. She participated in a number of R&D projects in Belgium and Bulgaria, authored and co-authored about 40 scientific papers in Bulgarian and international journals, as well as several book chapters.

Vedrin Jeliazkov (VJ): Vedrin received a M.Sc. in Computer Science from the University Paris VII - Denis Diderot, Paris, France in 1998. From 1996 to 1998 he worked for the R&D department of Electricité de France, Clamart, France, as a software developer, and was responsible for the design of quality assurance tests. From 2001 to 2002 Vedrin had been employed by the European Commission as an advisor to the director of "Essential Technologies and Infrastructures" at the Information Society Directorate-General. From 2003 to 2007 he was researcher at the Bulgarian Academy of Sciences and network engineer at the Network Operating Centre of the Bulgarian National Research and Education Network. Vedrin is one of the founders and owners of Ideaconsult Ltd, and is a full-time researcher at the company since 2007. He participated in a number of R&D projects in France, Belgium, and Bulgaria, authored ten research papers, co-authored one book and has given several talks in scientific conferences.

## Supplementary Material

Additional file 1 **Supporting Information**. Examples of accessing various AMBIT REST services via the cURL tool. Click here for file

Additional file 2 **Supporting Information**. RDF graph, representing two remote models, using the same training dataset. Click here for file
