# Supplementary material for: AMBIT RESTful web services: an implementation of the OpenTox application programming interface
Source: J Cheminform. 2011 May 16;3:18. doi: 10.1186/1758-2946-3-18 (PMC3120779; doi:10.1186/1758-2946-3-18)
Supplement: Additional file 1 — Supporting Information. Examples of accessing various AMBIT REST services via the cURL tool. [file 1758-2946-3-18-S1.DOC]

**Supporting Information: Examples of accessing various AMBIT REST services via the cURL tool**

**Chemical compounds**

Several Chemical MIME formats are supported, as well as the image/png format, which returns an image of the structure diagram of the compound. Thus, we avoid introducing custom syntax for format conversions, and instead use MIME types:

curl -H "Accept:chemical/x-daylight-smiles" http://apps.ideaconsult.net:8080/ambit2/compound/1

O=C

In the case of AMBIT services, the compounds are retrieved from the database backend, where they have been previously created by dataset or compound service POST commands, but an alternative implementation could simply be a wrapper for a public chemical structure database.

**Chemical compounds search**

Search results (by identifiers, similarity, or substructure) are also available as datasets:

curl -H "Accept:application/rdf+xml" http://apps.ideaconsult.net:8080/ambit2/query/compound/50-00-0/all

curl -H "Accept:application/rdf+xml" http://apps.ideaconsult.net:8080/ambit2/query/similarity?search=c1ccccc1O

curl -H "Accept:application/rdf+xml" http://apps.ideaconsult.net:8080/ambit2/query/smarts?search=a[Cl,Br,I,F]

**Datasets and features**

Datasets (*ot:Dataset* class) are managed by the dataset service *http://host:port/{service}/dataset*, which can be used to create new datasets (POST), update (PUT) and delete (DELETE) datasets. One option for creating a dataset is by uploading an *sdf* file of chemical compounds and properties by executing the following command:

curl -X POST -H 'Content-Type:chemical/x-mdl-sdfile' --data-binary @filename.sdf http://host:port/ambit2/dataset

The result of PUT and POST commands will be a dataset URI. Once a dataset is created, it could be retrieved in several supported formats.

The OpenTox Dataset class encapsulates compounds and their properties (feature) values. The RDF triples naturally allow to model binary relationships via the Subject-Predicate-Object construct (e.g. *molecular_weight has_value 200*). In order to model higher order relationships (e.g. CompoundX hasProperty molecular_weight with value 200) and more complex statements, two more classes have been introduced in the OpenTox resource ontology, namely *ot:FeatureValue* and *ot:DataEntry*. The *ot:FeatureValue* class encapsulates the relationship Feature – hasValue – Value. This is formally defined via the object property *ot:feature*, which links to the *ot:Feature* class, and the data property *ot:value*, which holds the value itself. This can be thought of as a cell in a table, where each cell contains not only the value, but a reference to the column header as well. This results in a flexible representation, not limited to tabular values. The *ot:DataEntry* class encapsulates the relationship *Compound* – *has values for* – *specific Features*. This can be thought as a row in a table, where the object property *ot:compound* specifies the *ot:Compound* resource, while the *ot:values* object property specifies all the cells (*ot:FeatureValue* instances), available in the data entry. A dataset consists of multiple instances of *ot:DataEntry*. The feature representation of the “Canc” field of the ISSCAN dataset, available via the AMBIT services and the OpenTox API, can be retrieved by executing the following command:

curl -H "Accept:application.rdf+xml" http://apps.ideaconsult.net:8080/ambit2/feature/21573

<rdf:RDF

xmlns:ot="http://www.opentox.org/api/1.1#"

xmlns:otee="http://www.opentox.org/echaEndpoints.owl#"

xmlns:dc="http://purl.org/dc/elements/1.1/"

xmlns="http://apps.ideaconsult.net:8080/ambit2/"

xmlns:rdf="http://www.w3.org/1999/02/22-rdf-syntax-ns#"

xmlns:owl="http://www.w3.org/2002/07/owl#"

xmlns:xsd="http://www.w3.org/2001/XMLSchema#"

xmlns:rdfs="http://www.w3.org/2000/01/rdf-schema#"

xml:base="http://apps.ideaconsult.net:8080/ambit2/">

<ot:Feature rdf:about="feature/21573">

<dc:creator>http://www.epa.gov/NCCT/dsstox/sdf_isscan_external.html</dc:creator>

<ot:hasSource rdf:resource="dataset/ISSCAN_v3a_1153_19Sept08.1222179139.sdf"/>

<owl:sameAs rdf:resource="http://www.opentox.org/echaEndpoints.owl#ENDPOINT_Carcinogenicity"/>

<ot:units></ot:units>

<dc:title>Canc</dc:title>

<rdf:type rdf:resource="http://www.opentox.org/api/1.1#NominalFeature"/>

</ot:Feature>

</rdf:RDF>

The feature representation of a subset of fields of the CPDBAS dataset, available via the AMBIT services and the OpenTox API, can be retrieved by executing the following command:

curl -H "Accept:application.rdf+xml" http://apps.ideaconsult.net:8080/ambit2/dataset/10/feature

<rdf:RDF

xmlns:ot="http://www.opentox.org/api/1.1#"

xmlns:otee="http://www.opentox.org/echaEndpoints.owl#"

xmlns:dc="http://purl.org/dc/elements/1.1/"

xmlns="http://apps.ideaconsult.net:8080/ambit2/"

xmlns:rdf="http://www.w3.org/1999/02/22-rdf-syntax-ns#"

xmlns:owl="http://www.w3.org/2002/07/owl#"

xmlns:xsd="http://www.w3.org/2001/XMLSchema#"

xmlns:rdfs="http://www.w3.org/2000/01/rdf-schema#"

xml:base="http://apps.ideaconsult.net:8080/ambit2/">

<ot:NominalFeature rdf:about="feature/21610">

<ot:hasSource rdf:resource="dataset/CPDBAS_v5d_1547_20Nov2008.sdf"/>

<owl:sameAs rdf:resource="http://www.opentox.org/echaEndpoints.owl#ENDPOINT_Carcinogenicity"/>

<dc:title>ActivityOutcome_CPDBAS_Rat</dc:title>

</ot:NominalFeature>

<ot:Feature rdf:about="feature/21607">

<ot:hasSource rdf:resource="dataset/CPDBAS_v5d_1547_20Nov2008.sdf"/>

<owl:sameAs rdf:resource="http://www.opentox.org/api/1.1#IUPACName"/>

<dc:title>STRUCTURE_ChemicalName_IUPAC</dc:title>

</ot:Feature>

<ot:NumericFeature rdf:about="feature/21603">

<ot:hasSource rdf:resource="dataset/CPDBAS_v5d_1547_20Nov2008.sdf"/>

<owl:sameAs rdf:resource="an-ontology-entry-representing-molecular-weight"/>

<dc:title>STRUCTURE_MolecularWeight</dc:title>

</ot:NumericFeature>

A simplified representation of an OpenTox Dataset is provided in Table 1.

| Data Entries | ot:compound | ot:values | | | |
| --- | --- | --- | --- | --- | --- |
|  |  | ot:FeatureValue | | ot:FeatureValue | |
|  |  | ot:feature | ot:value | ot:feature | ot:value |
| ot:dataEntry | /compound/147678 | /feature/22114 | 2.74 | … | … |
| ot:dataEntry | /compound/2146 | /feature/22114 | 1.59 | … | … |

Table 1. A simplified representation of an OpenTox Dataset.

**Data processing, model building, and predictions**

A data processing algorithm can be launched by executing the following command:

curl -H "Accept:supported-mime-format" -X POST -d 'dataset_uri=http://host:port/{service}/dataset/{id}' -d \ 'dataset_service= http://host:port/{service}/dataset/{id}' http://host:port/{service}/algorithm/{id}

The final result will be a URI of a dataset with the predictions. {id} is a template for the object identifier. It could be numeric or string, depending on the implementation. “supported-mime-format” is text/uri-list or RDF mime type.

A model building algorithm can be launched by executing the following command:

curl -H "Accept:supported-mime-format" -X POST -d 'dataset_uri=http://host:port/{service}/dataset/{id}' -d \ 'dataset_service= http://host:port/{service}/dataset/{id}' -d ‘prediction_feature='dataset_service= \ http://host:port/{service}/feature/{id}' http://host:port/{service}/algorithm/LR

The final result will be a URI of the new model. A POST command to the model URI, with a dataset or compound URI as input parameter, can be later used to calculate predictions:

curl –H “Accept:supported-mime-format” –X POST –d ‘dataset_uri=http://host:port/{service}/dataset/{id}’ –d \ 'dataset_service= http://host:port/{service}/dataset/{id}' http://host:port/{service}/model/{id}

The result will be a URI of a dataset, containing the predictions.

**A uniform approach to validation and reporting**

OpenTox includes a validation API, independent of the model building and predictions. While AMBIT services do not provide implementation of this API, models from any installation of AMBIT services can use remote OpenTox validation and reporting services, as explained in <http://opentox.org/dev/apis/api-1.2/Validation>, and according to the following example:

curl -d test_dataset_uri=http://host:port/ambit2/dataset/{id} -d \ prediction_feature=http://host:port/ambit2/feature/{id} -d model_uri=http://host/ambit2/model/{id} \ http://opentox.informatik.uni-freiburg.de/validation

**Ontology service**

An ontology service is available at <http://ambit.uni-plovdiv.bg:8082/ontology> and <http://apps.ideaconsult.net:8080/ontology>. A local copy can be installed by deploying *ontology.war* into a servlet container.

Resources are registered into the ontology service via a POST command, as shown in the following examples:

curl –X POST –d ”uri=http://host:port/ambit2/model/{id}” http://host:port/ontology

curl –X POST –d ”uri=http://host:port/ambit2/algorithm/{id}” http://host:port/ontology
